# Microbiome Analysis of Mucosal Ileoanal Pouch in Ulcerative Colitis Patients Revealed Impairment of the Pouches Immunometabolites

**DOI:** 10.3390/cells10113243

**Published:** 2021-11-19

**Authors:** Orazio Palmieri, Stefano Castellana, Giuseppe Biscaglia, Anna Panza, Anna Latiano, Rosanna Fontana, Maria Guerra, Giuseppe Corritore, Tiziana Latiano, Giuseppina Martino, Tommaso Mazza, Angelo Andriulli, Francesco Perri, Fabrizio Bossa

**Affiliations:** 1 Division of Gastroenterology, Fondazione IRCCS “Casa Sollievo della Sofferenza”, 71013 San Giovanni Rotondo, Italy; giuseppe.biscaglia@gmail.com (G.B.); a.panza@operapadrepio.it (A.P.); a.latiano@operapadrepio.it (A.L.); r.fontana@operapadrepio.it (R.F.); mariellaguerra90@gmail.com (M.G.); giuseppe.corritore@gmail.com (G.C.); tiziana.latiano@gmail.com (T.L.); giuseppina.martino@libero.it (G.M.); a.andriulli50@gmail.com (A.A.); f.perri@operapadrepio.it (F.P.); 2 Bioinformatics Unit, Fondazione IRCCS “Casa Sollievo della Sofferenza”, 71013 San Giovanni Rotondo, Italy; s.castellana@operapadrepio.it (S.C.); t.mazza@operapadrepio.it (T.M.)

**Keywords:** ulcerative colitis (UC), IBD, IPAA, pouch, pouchitis, microbiota

## Abstract

The pathogenesis of ulcerative colitis (UC) is unknown, although genetic loci and altered gut microbiota have been implicated. Up to a third of patients with moderate to severe UC require proctocolectomy with ileal pouch ano-anastomosis (IPAA). We aimed to explore the mucosal microbiota of UC patients who underwent IPAA. Methods: For microbiome analysis, mucosal specimens were collected from 34 IPAA individuals. Endoscopic and histological examinations of IPAA were normal in 21 cases, while pouchitis was in 13 patients. 19 specimens from the healthy control (10 from colonic and 9 from ileum) were also analyzed. Data were analyzed using an ensemble of software packages: QIIME2, coda-lasso, clr-lasso, PICRUSt2, and ALDEx2. Results: IPAA specimens had significantly lower bacterial diversity as compared to normal. The microbial composition of the normal pouch was also decreased also when compared to pouchitis. *Faecalibacterium prausnitzii*, *Gemmiger formicilis*, *Blautia obeum*, *Ruminococcus torques*, *Dorea formicigenerans*, and an unknown species from *Roseburia* were the most uncommon in pouch/pouchitis, while an unknown species from *Enterobacteriaceae* was over-represented. *Propionibacterium acnes* and *Enterobacteriaceae* were the species most abundant in the pouchitis and in the normal pouch, respectively. Predicted metabolic pathways among the IPAA bacterial communities revealed an important role of immunometabolites such as SCFA, butyrate, and amino acids. Conclusions: Our findings showed specific bacterial signature hallmarks of dysbiosis and could represent bacterial biomarkers in IPAA patients useful to develop novel treatments in the future by modulating the gut microbiota through the administration of probiotic immunometabolites-producing bacterial strains and the addition of specific prebiotics and the faecal microbiota transplantation.

## 1. Introduction

Ulcerative colitis (UC) is a chronic inflammatory disease of unknown etiology that affects the colon and rectum. The exact pathogenesis of UC is still unknown even if multiple factors including genetic background, environmental factors, altered gut microbiota, and dysregulated mucosal immune have been implicated [1].

The therapeutic approach in UC patients mainly depends on the severity of the disease and the extent of the inflammation. Up to a third of patients with moderate to severe UC still requires colectomy, due to therapy failure or to the development of dysplasia [2]. The gold standard surgical procedure in patients with UC is the proctocolectomy with ileal pouch ano-anastomosis (IPAA) [3]. However, up to half of the patients after IPAA develop inflammation of this reservoir, named pouchitis, which represents the most frequent complication [4]. The etiology of pouchitis is not entirely understood, although several risk factors have been proposed, such as a previous extensive UC or the presence of backwash ileitis, concomitant primary sclerosing cholangitis, non-steroidal anti-inflammatory drugs use, and positivity for pANCA, ASCA, and Anti-CBir1 [5,6]

From a genetic perspective, some polymorphisms hitting IL-1RA, TNFalpha [7], TLR9 [8], and NOD2/CARD15 were associated with an increased risk of pouchitis [9]. All of these studies are limited by the small numbers of enrolled patients and conflicting findings. Among the triggering factors, several lines of evidence strongly support the role of the microbiota, though it is not understood if the microbial dysbiosis could be the cause or the result of the pouch inflammation. In IBD patients with acute pouchitis, antibiotics are the mainstay of treatment [10], and the use of VSL#3 both for the maintenance of antibiotic-induced remission and for the prevention of pouchitis has been largely investigated [11].

Several microbial studies reviewed by Segal JP and colleagues [12] carried out in faecal and mucosal specimens have shown dysbiosis in patients with pouchitis, although there was no consensus about the abundance of specific species. At the faecal level, the most consistent finding concerning disease activity and the hallmarks of dysbiosis was a reduction of certain protective bacteria such as *Clostridiales* and an increase of inflammatory ones, specifically certain species belonging to *Enterococcus* or *Enterobacteriaceae*, which is associated with pouch inflammation in patients with UC [13].

To date, a specific microbial signature has not been identified probably due to the presence of a highly heterogeneous study design, sampling techniques, and analysis that released contradictory and inconclusive findings. Moreover, until now, no information is available about the functional composition of the discovered microbial communities.

Although there is a general agreement that the microbiota of both pouch and pouchitis is unstable over time, scarce data are available to figure out if the microbial pouch community is more ileal-like or tends to shift to be more colon-like [14,15].

### Study Outcomes

The study aimed to explore the microbiota resident at the mucosal level of UC patients with normal and inflamed pouches sequencing the 16S-rRNA by Next-Generation Sequencing technology. The hypothesis was that the mucosal microbiota composition of UC patients who underwent IPAA surgery could be associated with a higher risk of developing microbial dysbiosis and inflammation of the pouch, thereby opening new avenues to prevent and treat this complication. Our secondary aim was to study the microbial community of both bowel localizations involved in creating IPAA to determine if the pouch’s microbiota is more ileal-like or colon-like.

## 2. Methods

### 2.1. Patients

Multiple biopsies of the ileoanal pouch were taken from patients with IPAA who underwent endoscopy at the IBD Unit at Fondazione IRCCS ‘Casa Sollievo della Sofferenza’ Hospital, San Giovanni Rotondo (Italy) between 2013 and 2019. The control group consisted of healthy non-IBD and non-hospitalized individuals (HC—healthy controls). All specimens were snap-frozen in liquid nitrogen and stored at −80 °C before being processed.

The definition of pouchitis was determined using the Pouchitis Disease Activity Index (PDAI) that includes clinical, endoscopic, and histologic criteria [16].

### 2.2. Laboratory Procedures

DNA was extracted using the AllPrep PowerViral DNA/RNA Kit (Qiagen, Hilden, Germany) following the manufacturer’s recommendations. The DNA quantity was examined for each sample using a NanoDrop ND-1000 spectrophotometer (Thermo Fisher Scientific, Waltham, MA, USA). Microbial diversity analysis in the mucosal specimens was studied by sequencing the amplified V3 to V4 hypervariable region of the 16S rRNA gene on the MiSeq platform (Illumina, San Diego, CA, USA). PCR primers and conditions followed the Illumina 16S Metagenomic Sequencing Library preparation guide (Part # 15044223 Rev.B) [17] with the following exceptions: for the initial 16S PCR, the process was performed using Taq Phusion High-Fidelity (Thermo Fisher Scientific, Waltham, MA, USA) in 25 µL reaction volumes and 25 cycles.

The amplicons were purified using AMPure XP beads (Beckman Coulter, Pasadena, CA, USA). The ligation of the dual indexing adapters was performed in the presence of Nextera XT Index Primer 1 and Primer 2 (Illumina, San Diego, CA, USA), Taq Phusion High-Fidelity (Thermo Fisher Scientific, Waltham, MA, USA), and 5 μL purified DNA according to the manufacturer’s instructions. The products were purified using AMPure XP beads to create the final cDNA library. The library’s concentration and fragment size were measured using a fluorometric-based system (Qubit dsDNA BR Assay System; Thermo Fisher Scientific, Waltham, MA, USA) and the Agilent 2200 TapeStation Bioanalyzer (HS D1000 ScreenTape Assays; Agilent Technologies, Palo Alto, CA, USA), respectively. Equal amounts of cDNA libraries were pooled, denatured with NaOH, diluted with hybridization buffer to 7 pM following the Illumina protocol, and spiked with 20% PhiX (Illumina, San Diego, CA, USA). The libraries were loaded into a flow cell V2 (500 cycles) by paired-end sequencing (2 × 250) (Illumina, San Diego, CA, USA) and sequenced with the MiSeq sequencing instrument System (Illumina, San Diego, CA, USA) according to the manufacturer’s recommendations.

### 2.3. Bioinformatics and Statistical Sata Analyses

**Figure 1:**
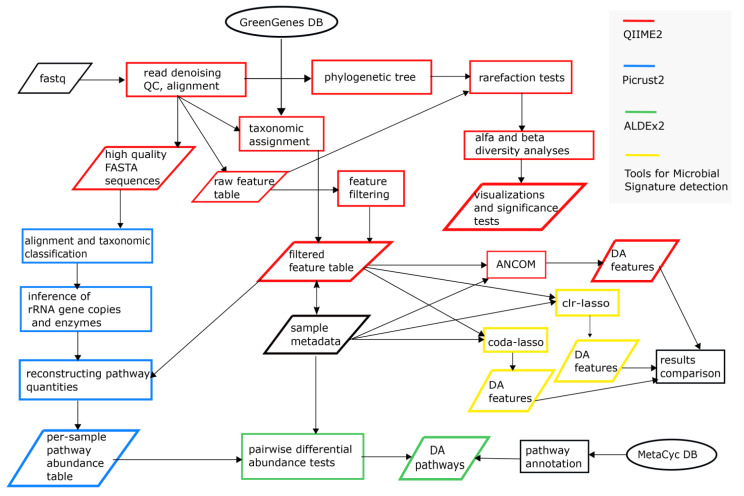
Analytical workflow of the study.

The analytical workflow (Figure 1) was composed of popular open-source tools and R packages [18] for metagenomics studies. Although simpler than the shotgun sequencing approach, 16S rRNA-based metagenomics displays several experimental and computational challenges [19]. In order to strengthen our results, we carried out redundant analytical strategies (e.g., in the identification of differential abundant microbial taxa) and accurately tuned tool parameters (e.g., rarefaction tests, feature table, and filtering), taking into account the compositional nature of metagenomic data. Several bioinformatics tools have been developed and carefully evaluated to tackle these aspects [20,21,22,23].

Sample reads were demultiplexed and subsequently analyzed using the QIIME2 v.2020.8 suite [24]. The initial step, which includes read quality checking and filtering, removal of identical reads (dereplication), chimeric reads identification, paired-end reads joining, and sequence clustering, was performed using DADA2 [25]; a raw feature table was then generated. De-noised sequences were also aligned and a 16S phylogenetic tree was constructed using one of the phylogenetic modules in QIIME2 via FastTree. Feature taxonomic classification was obtained using the QIIME2 embedded Naïve Bayes fitted classifier, pre-trained on the more recent Greengenes reference database (ver. 13.8) [26].

All samples were then rarefied following the indication of the Feature Table produced by the previous step, and any possible loss of information was ruled out by examination of the rarefaction plots. Rarefaction curve analysis of the obtained data was used to estimate the completeness of microbial communities sampling. Subsequently, we computed several alpha (Shannon’s diversity index [27], Number of Observed Features, Faith’s Phylogenetic Diversity [28], and Pielou’s evenness [29]) and beta diversity metrics (Jaccard distance [30], Bray–Curtis distance [31], unweighted UniFrac, and weighted UniFrac distances [32]) and generated Principal Coordinates Analysis (PCoA) plots using EMPeror application [33] for each beta diversity metric. Group significance between alpha and beta diversity indexes was calculated with QIIME2 plugins using the Kruskal–Wallis test and permutational multivariate analysis of variance (PERMANOVA), respectively. Differential abundance between groups on each taxonomic level was tested with ANCOM, “Analysis of Composition of Microbiomes” [34], with a taxa-wise correction for multiple testing. The ANCOM procedure compares the relative abundance of a taxon between two groups by performing statistical tests on data transformed by an additive log-ratio (Aitchison’s log-ratio) of the abundance of given taxon versus the abundance of all other taxa, individually. The W-value generated by the ANCOM method is a count of several sub-hypotheses (log-ratios of taxa relative abundances) that were detected to be significantly different across tested groups for a given taxon. We implemented the QIIME2-ANCOM plugin on two filtered feature tables, collapsed, respectively, at the species and genus level. Filters applied on the original QIIME2 Feature Table were inspired from Estaki M et al. [24]; we removed contaminant sequences (i.e., of mitochondrial/chloroplast origin) and features that appeared in less than 5 samples (i.e., around 10% of the total considered samples, 53) and those with less than 20 counts (across all samples). The QIIME2 commands pipeline is available within Supplementary File S4. The cladogram for the ANCOM results was produced using Graphlan Package v1.1.3 [35] and the genus-collapsed QIIME2 phylogeny as input.

We also implemented two additional compositional data analysis algorithms to define which features were significantly unbalanced among groups (normal pouch, pouchitis, HC colon, and HC ileum). We opted to implement the coda-lasso [36] and clr-lasso [37] methods as described in https://malucalle.github.io/Microbiome-Variable-Selection/ (accessed on 1 April 2021) to possibly detect a sort of microbial signature between groups. Thus, according to sample names and groups, we split the global feature table into seven sub-parts and applied both methods to all binary comparisons (e.g., pouch samples vs. pouchitis samples and pouch vs. HC colon). We considered these methods because they have been adequately conceived for sparse and compositional data (such as microbiomes); their results have been consistent and overlapping; they are not as computationally intensive as other methods; and they can easily be implemented in an R statistical environment. We generated a custom identifier for each feature within the feature table to facilitate the results interpretation, as shown in Supplementary File S2. Further details on group comparisons, methodological settings, and outcomes are in Supplementary File S2.

We determined our samples’ genetic and functional content by utilizing the PICRUSt v2.3.0 b software [38] and filtered the feature table and “representative sequences (rep-seqs.qza file)” using the QIIME2 pipeline. Briefly, rep-seqs.qza was converted into a FASTA format; these 16S sequence variants were then aligned and mapped to a maximum-likelihood-based phylogenetic tree. Then, PICRUSt2 allowed the prediction of the 16S gene content and gene families content for each mapped sequence on the tree. In the final step of the PICRUSt2 pipeline, two tables of predicted counts were produced: the former was relative to the per-sample enzyme content, while the latter showed the inferred pathway abundance per sample, named by their MetaCyc v24.5 [39] Identifiers.

In addition, we carried out a differential abundance analysis on the PICRUSt2 predicted abundances (unstratified pathway abundances, see Supplementary File S3 for refined and Supplementary File S4 for raw results), through a compositional data analysis tool, ALDEx2 [40]. Analogously to the binary comparisons for the microbial selection, the pathway abundance table was split according to the sample groups, and the ‘aldex’ command for pairwise analyses (for example, in the pouch vs. pouchitis samples comparison) was run. From the raw ALDEx2 output, we considered “significantly differentially abundant” the pathways with an estimated “effect” of ≥1.5 (absolute value, as suggested by the authors of ALDEx2).

## 3. Results

### 3.1. Clinicopathologic Characteristics

A total of 34 mucosal biopsy specimens were collected from UC patients with an ileal pouch, 21 of them with normal endoscopy and histology, and 13 with pouchitis. In addition, 19 specimens pinched from healthy controls (HC) were analyzed; 10 of them with normal colonic mucosa (5 female, mean age at recruitment 47.1 ± 16.2) and 9 with normal ileum (5 female, mean age at recruitment 36.2 ± 14.8). The general characteristics of the IPAA UC participants are given in Table 1.

### 3.2. 16S rRNA V3-V4 Region Sequencing Results

After the QIIME2 Quality Control procedure, we obtained a sample-specific yield ranging from 1953 to 75205 good quality reads. Given the raw feature table and the corresponding phylogenetic tree, we ran several rarefaction tests with different read depth cutoffs (see Supplementary Files S1 and S4 and Table S1.1) to establish an optimal sampling depth with a minimal sample loss. Rarefaction at a depth of 4910 determined the exclusion of only two poorly sequenced samples (a patient with pouchitis and an HC patient with ileal localization with sample identifiers “526TV” and “CON618”, respectively, in Supplementary File S1, Table S1.1) while guaranteeing the stable distribution of three alpha diversity metrics in all of the four investigated groups.

### 3.3. Microbial Diversity and Community Analyses

Some significant differences in alpha and beta diversities emerged between controls and IPAA UC patients. In detail, alpha diversity was evaluated by analyzing four metrics (Shannon Entropy, Pielou’s evenness, Number of Observed Features, and Faith’s Phylogenetic Distance) and comparing group-specific distributions through Kruskal–Wallis tests, global and pairwise. Table 2 reports q-values for pairwise group comparisons, while whole results and boxplots for diversity analyses are reported in Supplementary File S1, Sections S1.4 and Section S1.5.

**Figure 2:**
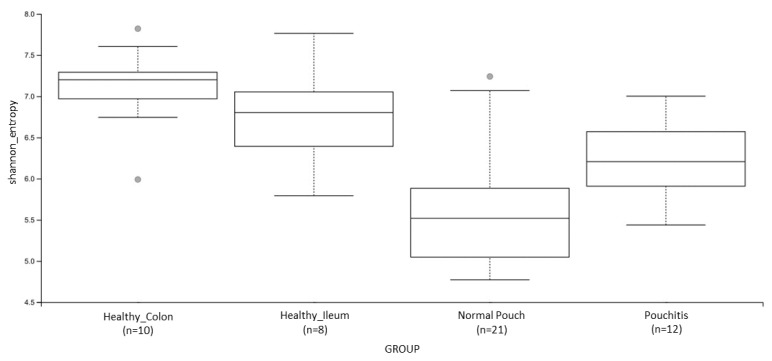
Boxplot for group-specific Shannon’s entropy distributions. See Table 2 and Supplementary File S1 for details. Kruskal–Wallis (all groups): H = 25.1; *p* value = 0.000015.

When we compared normal pouch vs. pouchitis groups, Shannon’s entropy (Figure 2) and Pielou’s evenness had significantly different results (both *q*-values = 0.01), being that normal pouch communities are characterized by a lower diversity and a lower “equitability” (i.e., taxa are less uniformly abundant in normal pouches). No significant differences emerged when qualitative measures, i.e., the “number of observed features” (a raw estimate of species richness) and “Faith’s PD” (based on phylogenesis) were taken into account.

The microbiota results were significantly dissimilar for alpha indexes, namely Shannon’s diversity, Pielou’s evenness, and the Number of Observed Features when patients with a normal pouch and those with pouchitis were compared to colon specimens of HC. Taking into account the microbiota from the normal pouch and pouchitis vs. HC ileum groups, those with a normal pouch were statistically significantly dissimilar for what concerns Shannon’s diversity, Pielou’s evenness, and Faith’s Phylogenetic Distance distributions (*q*-values < 0.005, < 0.001 and < 0.02, respectively), while those with pouchitis were statistically different for evenness and Faith’s Phylogenetic indexes (*q* values < 0.01 and < 0.02, respectively). Normal pouch samples exhibited a lower microbial diversity and equitability (i.e., taxa were not uniformly abundant) than the other investigated environments.

We also compared specimens of healthy samples (colon vs. ileum). Although we were dealing with minimal sample sizes, we detected a significantly higher species richness within colon samples, as shown by the two qualitative metrics, Faith’s PD and the number of observed features. Interestingly, Faith’s PD values for the eight biopsies from the ileum of healthy samples were very low and homogeneous, suggesting that strictly phylogenetically related taxa mainly constitute the ileal microbial composition.

**Figure 3:**
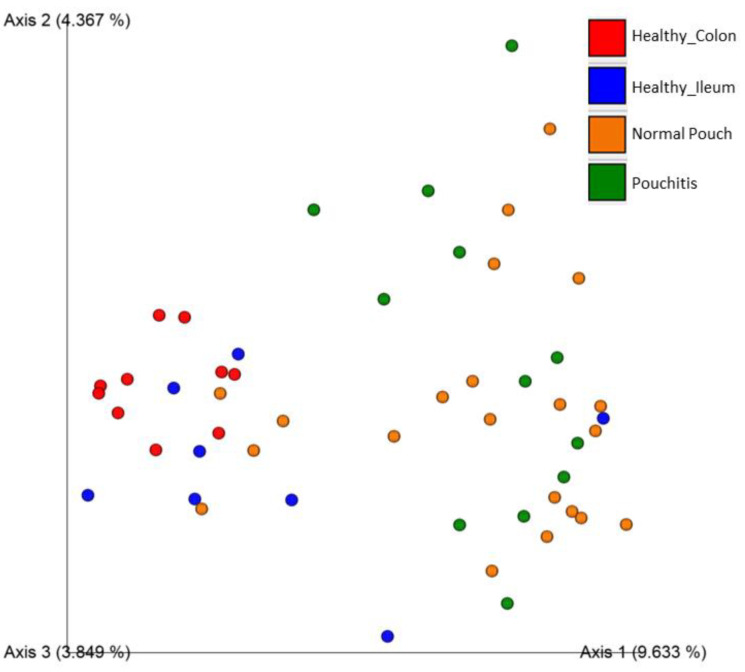
Principal Coordinates Analysis Plot for Healthy Colon (red), Healthy Ileum (blue), Normal Pouch (yellow), and Pouchitis (green) sample groups; distances across samples are calculated through Jaccard Index. More details are in Supplementary File S1.

We also compared four popular Beta dissimilarity metrics (Bray–Curtis, Jaccard, Unweighted Unifrac, and Weighted Unifrac) through the PERMANOVA pairwise test (at 999 permutations) and visualized sample dissimilarity through Emperor Plots (Supplementary File S1). A comparison between the groups of normal pouch and pouchitis showed that there is not any significant variability, in terms of species richness and abundance, between the two environments (only for weighted UniFrac dissimilarity a slightly significant difference was found; *q*-value = 0.048). When we compared the microbiota of patients with a normal pouch to those of a healthy ileum or healthy colon, the differences among the samples were significantly different for most of all the considered metrics, as shown in Table 3 and for the Jaccard distance (Figure 3). Visualizations of beta diversity measures through Emperor PCoA plots confirmed that normal pouch and pouchitis samples were overlapping.

After analyzing the global microbial diversity through alpha and beta metrics, we implemented the QIIME2 ANCOM module to analyze the taxa-relative abundance across sample groups. After the filtering step (see Methods), we obtained a feature count matrix of 474 rows (features) and 53 columns (samples).

The ANCOM analysis collapsed at the genus level revealed organisms named *Faecalibacterium* (ANCOM W statistics = 48) and *Gemmiger* (W = 47), showing significant differential abundances in healthy (ileum/colon) localizations as compared to normal or inflamed pouches. Furthermore, a taxon belonging to the *Enterobacteriaceae* family (W = 48) resulted in being more abundant in both cohorts of UC patients (Supplementary File S1).

**Figure 4:**
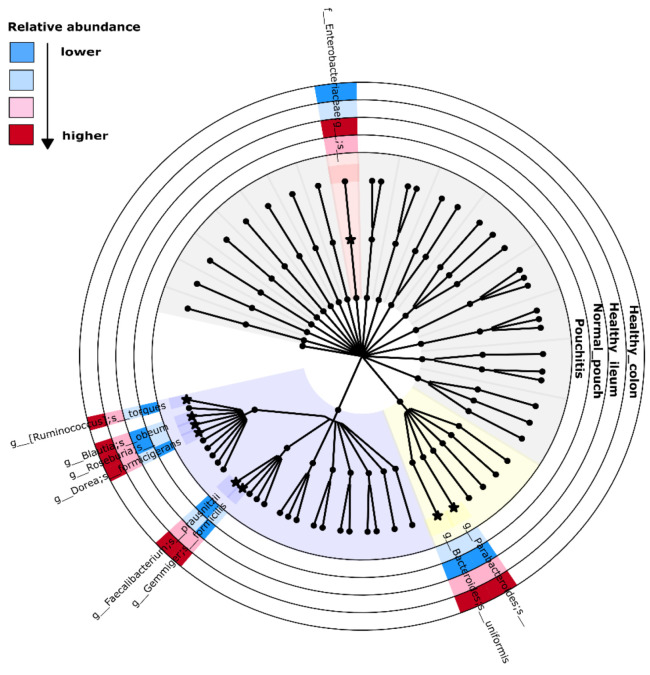
Cladogram for ANCOM significant results. Concentric circles represent the four investigated groups. Cladogram has been generated by implementing Graphlan Package on the genus-collapsed taxonomy by QIIME2. Internal nodes: order and family; leaves: genus. Background colors: violet for *Clostridiales*, yellow for *Bacteroidales*, red for *Enterobacteriales*, and grey for remaining groups. Starred nodes and colored sections are relative to ANCOM-relevant taxa: extended taxonomic name is adjacent to nodes, while color interpretation is shown in the top-left part.

We also performed an ANCOM analysis at the species level, finding significant differences in relative abundances for *Faecalibacterium prausnitzii* (W = 65), *Gemmiger formicilis* (W = 64), *Blautia obeum* (W = 53), *Ruminococcus torques* (W = 53), *Dorea formicigenerans* (W = 43), *Bacteroides uniformis* (W = 42), and two unannotated species belonging to the genera named *Roseburia* (W = 61) and *Parabacteroides* (W = 50). They resulted in more abundance in healthy colon and ileum specimens than the normal pouch/pouchitis groups. The features mapped to the *Enterobacteriaceae* family resulted in being significantly abundant in the IPAA patient groups (W = 64) (Supplementary File S1, Section S1.6). Importantly, ANCOM is based on compositional log ratios between the relative abundances of two taxa and is performed on each taxon couple; the associated W statistics then indicate the number of times (more precisely, “sub-hypothesis”) that the relative abundance of taxon “A” is different from the ones of the other taxa. In the above-mentioned case of *Faecalibacterium prausnitzii*, the value of W = 65 indicates that its relative abundance is different from those of 65 other taxa along with the compared groups. Top-right dots in the ANCOM Volcano plots defined the significant features, while the percentile abundances tables showed the feature counts at 0%, 25%, 50%, 75%, and 100% of the samples within each group. A summarized version of the main ANCOM results is given in the Figure 4 cladogram.

### 3.4. Microbial Signature from Pairwise Group Comparisons

Differential microbial abundance between groups was performed using two compositional data analysis (CoDA) approaches, namely coda-lasso and clr-lasso. Both strategies were described in Supplementary File S2; the “Results” sheet provides a list of the selected variables (i.e., the features represented by custom identifiers) together with their corresponding correlation coefficients and methodological details, such as the “method name,” lambda penalization parameter, number of identified variables, the proportion of explained deviance, and group class (encoded as “1” or “0”, as defined in the sheet “Pairwise_comparison”).

**Figure 5:**
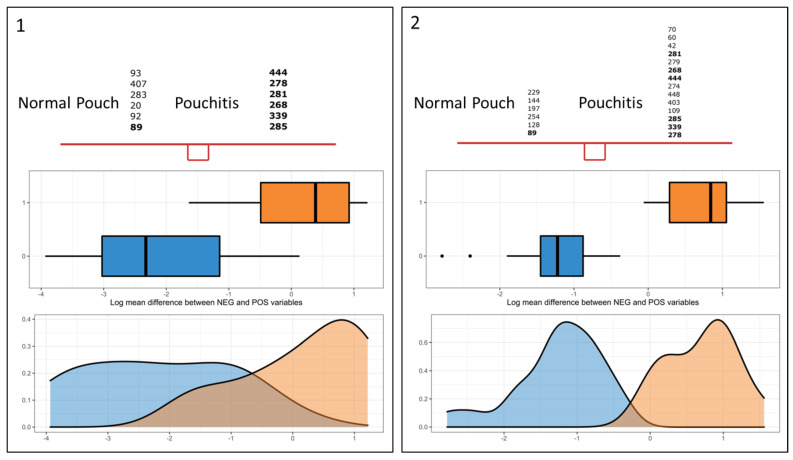
Differential microbial abundance between the normal pouch and pouchitis groups by using two compositional data analysis (CoDA) approaches, namely coda-lasso (panel **1**) and clr-lasso (panel **2**). The “selbal-like” plots show the feature identifiers for the clr-lasso- and coda-lasso-selected taxa that have greater abundance in “class 1” samples, i.e., the pouchitis samples (id in “A” list) and those more abundant in “class 0” samples (normal pouch, identifiers in “B” list). Bold numbers in the two top panels represent common features between methods; identifiers are ordered according to their absolute coefficients (lowest to highest and in top-down direction). Middle and bottom panels show, respectively, boxplots of the scores (or balances) as calculated for each sample within each sample group and density plots of such balances. “Balance” resumes the mean log-transformed abundances of the taxa in the comparing groups.

We observed that 15 features, which can be collapsed to 10 different taxonomic classifications, were found in common between both methods: a feature “X” has been considered “common” when its relative abundance was found to be significantly associated with the same group class according to both techniques, although relative regression coefficients could differ. Our main task was to find a microbial signature in the normal pouch/pouchitis system, i.e., a subset of taxa whose relative abundances significantly discriminated between the two states. Figure 5 (“selbal-like” plots) shows the feature identifiers for coda-lasso (panel 1) and clr-lasso (panel 2) selected. Taking into consideration the common consistent features (bold identifiers in Figures) and the QIIME2 taxonomic classification, the “pouchitis—normal pouch” microbial signature included the following taxa (sheet “Results interpretation”, Supplementary File S2): genus *Streptococcus* (id 285), *Propionibacterium acnes* (id 339 and 444), genus *parabaococcus* (id 268), *Clostridium ramosum* (id 281), and a feature associated to *Clostridiales* (id 278), which were significantly associated with pouchitis, while a feature associated with the *Enterobacteriaceae* family (id 89) exhibited greater abundance in the normal pouch. Interestingly, this unknown group of *Enterobacteriaceae* has also been evidenced by the ANCOM approach, resulting in a greater abundance in most samples from the normal pouch cohort.

Pairwise variable selection through clr-lasso and coda-lasso evidenced taxa that were somehow consistent with the ANCOM results. *Faecalibacterium prausnitzii* (id 234 and 275, Supplementary File S2) resulted in being more abundant in normal samples (ileum and colon) than the pouch environments of the IPAA patients. This species was found to be differentially abundant through ANCOM (Supplementary File S1). *Gemmiger formicilis* (id 142 and 307) and genus *Roseburia* (id 2 and 193) resulted in lower abundances in the patients’ groups (pouch and pouchitis) than when compared to the controls’ (colon and ileum). ANCOM analyses (at genus and species level) also identified these features as differentially abundant, although across the four sample groups.

*Parabacteroides* spp. (id 116) exhibited negative coefficients in both the “normal pouch vs. ileum” and “pouchitis vs. ileum’’ comparisons, thereby indicating a lower abundance in the pouch environment with respect to the ileal anatomical trait. Both variable selection methods returned several other features for all the considered pairwise group comparisons, as listed in Supplementary File S2. The methods have independently detected a total of 157 non-unique features, while 48 of them (30%) were in common.

### 3.5. Differential Analysis of Inferred Microbial Pathways

A total of 1671 gene functions (defined by Enzyme Commission identifiers) and 350 metabolic pathways (defined by MetaCyc identifiers) have been inferred using the PICRUSt’s pipeline on the 474 × 53 feature table (features × samples).

Given the PICRUSt “unstratified” (i.e., quantities are not stratified per sample) pathway quantity table, we identified differentially abundant pathways through pairwise group comparisons. Supplementary File S3 summarizes our comparative strategy together with the differentially abundant pathways retrieved by ALDEx2: the “ALDEx2_filtered” sheet shows a total of 29 (22 unique MetaCyc ids) differentially abundant items (filters described in Methods). We found relevant differences in pouchitis vs. healthy colon (Comparison 6), normal pouch vs. healthy colon (Comparison 3), and between control sample groups (Comparison 2).

Comparing normal pouch (21 samples) vs. healthy colon (10 samples) groups, we evidenced 10 pathways with a significantly higher abundance in the first group: fatty acid beta-oxidation (FAO-PWY), D-glucarate degradation I (GLUCARDEG-PWY), glucose, and glucose-1-phosphate degradation (GLUCOSE1PMETAB-PWY), a superpathway of polyamine biosynthesis I (POLYAMSYN-PWY), ubiquinol-related pathways (PWY-5855, PWY-5856, PWY-5857, PWY-6708, UBISYN-PWY), and dTDP-N-acetylthomosamine biosynthesis (PWY-7315).

Seven of the pathways mentioned above (FAO-PWY, GLUCOSE1PMETAB-PWY, PWY-5855, PWY-5856, PWY-5857, PWY-6708, and UBISYN-PWY) were also found to be over-abundant in the pouchitis group compared with in the healthy colon one. In addition, the microbial communities in pouchitis appeared to have a greater abundance of the 4-aminobutanoate degradation V pathway (PWY-5022). Supplementary File S3, the “ALDEx2_raw” sheet, provides the full ALDEx2 outcome. Although the comparison between colon and ileum samples (Comparison 2 in Supplementary File S3) is beyond the scope of this work, and although the sample size is reduced, we found 11 pathways to be significantly over-represented in the healthy colon compared with in the healthy ileum pool.

## 4. Discussion

Studies on animal models of chronic intestinal inflammation [41] and genome-wide association studies [42] suggest that a host–microbe interaction is often required to develop colitis in genetically susceptible hosts. It is known that susceptibility IBD genes encoding proteins required for innate or adaptive mucosal immune defense mechanisms indicate that the intestinal microbiota could be responsible for triggering the IBD disease. Strategies that modulate the gut microbiota, such as antibiotic treatment or elemental diets, are effective in reducing the inflammation in IBD patients [10].

It is well established that the microbiota composition of IBD patients is less diverse and differs from those of healthy individuals, a phenomenon often referred to as “dysbiosis,” widely described as an unbalance in gut microbiota associated with the disease. Despite many studies aimed at delineating how the gut microbiota is altered in UC patients who underwent the IPAA procedure [12], conflicting results were reported, partly due to differences in the study design and methodology used.

Our data demonstrate a high level of dysbiosis in IPAA patients, as highlighted by decreased microbial composition at non-parametric (Shannon and evenness) diversity and at beta diversity metrics. Here we highlighted that the microbiota from non-inflamed pouches and pouchitis has significantly lower entropy and less evenness and differ from those pinched from the normal colonic or ileal mucosa.

Interestingly, we pinpointed that the microbial composition of the normal pouch statistically also decreased when compared to those with pouchitis, showing that microbiota from the normal pouch is both weaker and not complex as compared to all the analyzed groups (i.e., including HC and patients with pouchitis).

The analysis of the composition of microbiomes (ANCOM test) showed two genera (*Faecalibacterium* and *Gemmiger*), both belonging to the *Ruminococcaceae* family, which resulted in their near absence in the pouch/pouchitis group while being over-represented in the colon/ileum of HC. On the other hand, an unknown genus from the *Enterobacteriaceae* family was over-represented in the pouch/pouchitis group as compared to in the controls.

As expected, at the species level, the *Faecalibacterium prausnitzii* and *Gemmiger formicilis* (*Ruminococcaceae*), *Blautia obeum*, *Ruminococcus torques*, *Dorea formicigenerans*, and one unknown species from the *Roseburia* genus (*Lachnospiraceae*) were the most uncommon in both the normal pouch and pouchitis groups, while an unknown species from the *Enterobacteriaceae* family was over-represented in the pouch/pouchitis group.

*Ruminococcaceae* and *Lachnospiraceae*, among *Firmicutes*, are known as species that hydrolyze starch and other sugars to produce butyrate and other SCFAs and have a pivotal role in maintaining the host’s well-being [43]. Our data are in accordance with the current literature, which reports a significant reduction in the *Ruminococcaceae* in patients with a UC pouch by imputing this taxon to the reduction to bile acid deficiency [44].

If little is known about *Gemmiger formicilis* and *Blautia obeum*, there is a lot of evidence that attributes a diagnostic and prognostic role to *Faecalibacterium prausnitzii*, since it is the most well-known butyrate-producing bacteria able to promote gut health [45]. A reduced level of this potentially ‘beneficial’ bacteria was found in one NOD2 positive sibling who developed pouchitis, while a normal *Faecalibacterium prausnitzii* level was found in his brother and mother, who remained pouchitis-free after IPAA surgery [46].

The *Lachnospiraceae* family belongs to four species, all decreased in IPAA UC patients. A reduction of two *Lachnospiraceae* genera, named *Blautia* spp. and *Roseburia* spp., have been previously identified [47] in the faeces of UC patients before surgery and were associated with a higher risk of pouchitis after IPAA. The *Ruminococcus torques* is known to degrade gastrointestinal mucin, and it was more frequently found in relative faeces of patients with CD [48]. Although little is known about *Dorea formicigenerans*, its decrease was found in the faecal samples of patients with rheumatoid arthritis [49] and is abundant in patients with Crohn’s disease in clinical remission [50]. At the species level, a further butyrate-producing organism, namely *Bacteroides uniformis*, was significantly reduced in our IPAA UC patients and was found also reduced in CD patients [51].

An unknown species from the *Parabacteroides* genus was found decreased in both IPAA UC patients; likewise, this genus was previously detected less frequently in patients with pouchitis compared to those without inflammation [52]. On the other hand, we found unknown species from the *Enterobacteriaceae* family in more abundance in IPAA UC patients. Noteworthy is that the enrichment of the iron-dependent pathogenic *Enterobacteriaceae* was detected in the stool of chronic pouchitis patients [53,54], highlighting an important role of the iron metabolism at the host–microbe interfaces since it presents a critical role in many aspects of the host’s normal physiology and in maintaining immunity [55]. In animal models of IBD, it has been shown that a high concentration of luminal iron or heme iron alters gut microbial communities, further exacerbating the bloom of *Enterobacteriaceae* and worsening colitis [56].

Further pairwise analyses highlighted 10 taxonomic features associated with IPAA patients. Of these, five, namely *Faecalibacterium prausnitzii*, *Gemmiger formicilis*, *Roseburia* spp., *Parabacteroides*, and the *Enterobacteriaceae* family, were in common with ANCOM. *Faecalibacterium prausnitzii*, *Gemmiger formicilis*, *Roseburia*, and *Parabacteroides* were confirmed in more abundance in healthy tissues as compared to in the microbial composition from the normal pouch and pouchitis groups, confirming their ‘beneficial’ bacterial role. On the other hand, features associated with the *Enterobacteriaceae* family resulted in more abundance in all the IPAA patient groups. More importantly, features belonging to *Enterobacteriaceae* resulted in more abundance in the normal pouch than when compared with pouchitis in both pairwise analyses through clr-lasso and coda-lasso. The pairwise analysis between the IPAA patient groups focuses on a further species, namely *Propionibacterium acnes* and *Clostridium ramosum*, as the most abundant features in pouchitis. *Propionibacterium acnes* produces SCFA and has been repeatedly proposed as the causative agent of sarcoidosis [57]. Although there is limited evidence, it was suggested of possible involvement in UC through the gut–brain–skin axis by influencing the host’s immune system. The *Clostridium ramosum* is a cause of several severe infections mainly in immunocompromised inpatients [58] and was identified in jejunal and ileal mucosal from CD patients [59].

An interesting result that emerges from our analysis of the prediction of functional genes among bacterial communities of IPAA patients revealed an important role of metabolites such as SCFA, butyrate, and amino acids, which could result in an imbalanced activation of the gut immune system. When both the IPAA cohorts were compared with those from the normal colon, increases of fatty acid beta-oxidation, glucose and glucose-1-phosphate degradation, and ubiquinol-related biosynthesis were observed. In addition, an increase of D-glucarate degradation I and the activation of biological mechanisms involved in polyamine and dTDP-N-acetylthomosamine biosynthesis were achieved only in the patients with a normal pouch. The identified pathways have in the bloom of the *Enterobacteriaceae* their top contributors [60]. More interestingly, when comparing the ileum of IPAA patients to normal ileum, no pathway was revealed. These results could outline that the microbial composition from IPAA patients is more similar to the ileal ones.

The sugars or biomolecules that the *Enterobacteriaceae* use for immediate energy and maybe to sustain the inflammatory response could derive from the D-glucarate degradation I (“GLUCARDEG-PWY”) [61,62] and from the glucose and glucose-1-phosphate degradation (“GLUCOSE1PMETAB-PWY”) [63]. A further pathway involved in providing energy and acetyl-CoA for cell growth is the fatty acid beta-oxidation (“FAO-PWY”) [64]. The aforementioned pathways are tightly coupled with ubiquinol-related pathways (PWY-5855”, “PWY-5856”, “PWY-5857”, “PWY-6708”, and “UBISYN-PWY) in the mitochondria. In these organelles, the products or metabolites released by their oxidation or degradation are used from the respiratory chain to produce energy and for a large variety of biosynthetic processes [65]. In addition, dTDP-N-acetylthomosamine (PWY-7315) are involved in the outer mitochondrial membrane biosynthesis [66]. Polyamine, synthesized by a superpathway of polyamine biosynthesis I (POLYAMSYN-PWY), are involved in many fundamental processes of cell growth, survival, immune response [67], and maintaining mucosal homeostasis in the intestine [68].

The global function of the gut microbiota in the modulation of both metabolism and immunity has been extensively studied [69,70], and its role at the service of “immunometabolism” is also becoming definite in IBD [71]. Remarkably, in the present study, many of the immunometabolite-producer bacteria and mucin-degrading bacteria were at lower abundance in IPAA patients, independently from the inflammatory state, while the *Enterobacteriaceae* (known as iron-produced bacteria) were more abundant in the normal pouch vs. pouchitis group, resulting in a deep dysbiosis and metabolites imbalance. Taken collectively, our data highlight an imbalance of a reduced number of beneficial symbionts bacterial vs. pathogenic, suggesting that bacterial products or endogenous synthetic/catabolic molecules contribute to an impairment of the immune response, a breakdown of the epithelial barrier, and the enhancement of the inflammatory processes, which imparts huge functional consequences to the host. We acknowledge some limitations of this study, such as the limited number of patients, although it is coherent with a single-center series. However, our computational strategy offered us the possibility to identify and quantify differences in microbial and functional content between IPAA and control samples. We experienced numerous technical challenges. First, we obtained a variable and generally low amount of high-quality reads, which pushed us to apply several rarefaction tests and feature/sample filtering criteria to obtain reliable diversity metrics and remove ultra-rare taxa. We also took advantage of several (even alternative) bioinformatic packages that, while providing valuable results, have their limitations. For example, functional differential abundance analyses were carried out on predicted gene quantities, and pathways were reconstructed through these quantities. Moreover, methods for microbial signature detection are powerful, but results are often not consistent, while parameter tuning and method implementation are complex procedures. We also hypothesized to validate our computational pipeline on public data; however, we did not retrieve consistent raw data (i.e., 16S sequencing reads or feature/OTUs tables from pouches) into specialized metagenomic resources [72,73].

## 5. Conclusions

Our findings showed specific bacterial signature hallmarks of dysbiosis and could represent bacterial biomarkers in IPAA UC patients useful for developing novel treatments in the future by modulating the gut microbiota through diet, the administration of probiotic immunometabolites-producing bacterial strains, the addition of specific prebiotics, and the faecal microbiota transplantation.

## Figures and Tables

**Table 1:** General characteristics of the IPAA UC patients.

	Normal Pouch	Pouchitis
	(*n* = 21)	(*n* = 13)
Gender (female %)	12 (57%)	4 (31%)
Mean age at recruitment (years SE)	48.2 (3.1)	49.3 (3.9)
Mean age at diagnosis (years SE)	33.9 (2.9)	34.4 (4.3)
Time from surgery to sample collection (years SE)	7.8 (1.2)	8.1 (1.7)
Mean age at surgery	40.8 (3.1)	41.6 (4.2)
Antibiotic previous month (% using)	0 (0%)	3 (25%)
Antibiotics ever (% used)	1 (4.8%)	3 (23%)

**Table 2:** Summary of pairwise group comparisons for four alpha diversity indexes. Benjamini and Hochberg corrected *p*-values (*q*-values) for Kruskal–Wallis tests are shown; “ns”: *q*-value > 0.05.

Comparison	Normal Pouch	Healthy Colon		Healthy Ileum		Healthy Ileum
	vs.	vs.		vs.		vs.
Measure	Pouchitis	Normal Pouch	Pouchitis	Normal Pouch	Pouchitis	Healthy Colon
Shannon’s Entropy	0.009	0.0004	0.004	0.005	ns	ns
Pielou’s evenness	0.013	0.003	0.035	0.001	0.011	ns
Number of Observed Features	ns	0.0005	0.049	ns	ns	0.013
Faith’s Phylogenetic Distance	ns	0.064	ns	0.022	0.022	0.008

**Table 3:** Summary of pairwise group comparisons for beta diversity measures. Benjamini and Hochberg corrected *p*-values (*q*-values) for PERMANOVA tests are shown; “ns”: *q*-value > 0.05.

Comparison	Normal Pouch	Healthy Colon		Healthy Ileum		Healthy Ileum
	vs.	vs.		vs.		vs.
Measure	Pouchitis	Normal Pouch	Pouchitis	Normal Pouch	Pouchitis	Healthy Colon
Jaccard similarity	ns	0.002	0.002	0.002	0.002	0.02
Bray–Curtis dissimilarity	ns	0.002	0.002	0.005	0.002	ns
Unweighted UniFrac dissimilarity	ns	0.008	0.063	0.004	0.004	0.002
Weighted UniFrac dissimilarity	0.048	0.04	0.04	0.05	0.04	0.04

## Data Availability

The data presented in this study are all available in the Supplementary Files.

## Supplementary Materials

The following are available online at https://www.mdpi.com/article/10.3390/cells10113243/s1 Supplementary File S1: sequencing statistics, taxonomical composition (feature counts), diversity and ANCOM analyses by QIIME2 pipeline, Supplementary File S2: microbial differential abundance analysis results by Coda_lasso and Clr_lasso methods, Supplementary File S3: pathway prediction and associated differential abundance measurement by Picrust2 and ALDEx2 tools, Supplementary File S4: full output for QIIME2 and Picrust2 implementations, used scripts/pipelines and sample metadata table.
