# Supplementary material for: Microbiome Analysis of Mucosal Ileoanal Pouch in Ulcerative Colitis Patients Revealed Impairment of the Pouches Immunometabolites
Source: Cells. 2021 Nov 19;10(11):3243. doi: 10.3390/cells10113243 (PMC8624401; doi:10.3390/cells10113243)
Supplement: Supplementary file 1 [file cells-10-03243-s001.zip › supplementary files/Supplementary_File_1_rev legends.pdf]

Supplementary File 1

1.1. Sequencing and alignment statistics.

First column: sample identifiers; "#non-chimeric" column indicates the number of high quality sequences per sample. Analysis performed by QIIME2 Suite (Supplementary File 4 for details).

Sample ID	# reads	# filtered	% filtered	#de-noised	#merged	% input merged	# non-chimeric	% non-chimeric
324RDA	92597	27025	29.19	26169	25565	27.61	6736	7.27
373DSE	239996	157973	65.82	155992	152061	63.36	30714	12.8
381BA	191270	141402	73.93	140200	138241	72.28	27350	14.3
396QL	103638	30946	29.86	29967	29239	28.21	6111	5.9
415CP	169072	123110	72.82	122070	120794	71.45	21865	12.93
432MF	185587	139226	75.02	137387	134685	72.57	19127	10.31
436CG	590780	420239	71.13	412225	375921	63.63	40434	6.84
438RNP	178562	134226	75.17	132505	126804	71.01	22106	12.38
451FV	228395	156059	68.33	153696	147943	64.78	29097	12.74
455CG	175890	126007	71.64	124786	122565	69.68	28539	16.23
469CS	77146	60214	78.05	59642	58395	75.69	8387	10.87
474SA	167525	53199	31.76	52379	51804	30.92	6620	3.95
475PMM	92711	71005	76.59	70294	69038	74.47	12715	13.71
476SS	107285	79954	74.52	78715	76924	71.7	14023	13.07
477CB	174201	119711	68.72	117258	110156	63.23	26789	15.38
502SC	96567	22286	23.08	21936	21439	22.2	5729	5.93
5064VC	187186	61004	32.59	60479	59300	31.68	8928	4.77
5062CV	119213	38821	32.56	38176	37583	31.53	6072	5.09
509CZG	655757	209431	31.94	207555	201329	30.7	39200	5.98
5161OE	438731	134020	30.55	132736	128673	29.33	27169	6.19
5165OE	416490	127614	30.64	126680	123076	29.55	26913	6.46
524SN	314371	97568	31.04	96857	95867	30.49	20657	6.57
525CM	743494	241900	32.54	238028	218771	29.42	18420	2.48
526TV	31610	9314	29.47	9085	8787	27.8	1953	6.18
529AE	135043	45777	33.9	45029	44329	32.83	7294	5.4
537AA	101961	33675	33.03	33132	32696	32.07	8892	8.72
539PM	190896	59109	30.96	58264	57351	30.04	12803	6.71
541TG	284090	95700	33.69	95084	94141	33.14	15803	5.56
607SL	382407	125985	32.95	124987	123430	32.28	20574	5.38
622PG	202172	61485	30.41	60936	59905	29.63	13090	6.47
648FM	61991	41134	66.35	39942	38842	62.66	8848	14.27
689TFP	86764	24243	27.94	23412	23114	26.64	5858	6.75
707LA	98716	32181	32.6	31659	31285	31.69	7920	8.02
709DPG	193336	118177	61.13	116559	112444	58.16	22257	11.51
CON015	255365	196315	76.88	194364	189279	74.12	24882	9.74
CON016	116845	87151	74.59	86199	84030	71.92	18466	15.8
CON017	94682	73412	77.54	72087	70313	74.26	12671	13.38
CON018	286955	232609	81.06	230557	224396	78.2	38565	13.44
CON019	127674	98086	76.83	96465	93725	73.41	16382	12.83
CON020	114042	89346	78.34	87928	85189	74.7	13839	12.14
CON021	120686	93008	77.07	91514	89329	74.02	14020	11.62
CON003	666215	530237	79.59	526656	514195	77.18	75204	11.29
CON004	109006	86310	79.18	84938	82175	75.39	14419	13.23
CON459	75827	22125	29.18	21662	20858	27.51	6046	7.97

CON617	239948	72273	30.12	71239	67608	28.18	9106	3.79
CON618	64539	19909	30.85	19216	17008	26.35	3549	5.5
CON629	117204	36621	31.25	35923	32953	28.12	4910	4.19
CON630	308617	98145	31.8	97182	94929	30.76	16574	5.37
CON631	264574	86432	32.67	84970	82165	31.06	9600	3.63
CON633	80548	27673	34.36	27173	26294	32.64	6281	7.8
CON638	131206	43103	32.85	42071	39673	30.24	6962	5.31
CON645	152227	49299	32.39	48045	45274	29.74	8246	5.42
CON007	206676	136952	66.26	134258	126255	61.09	25518	12.35

1.2. Feature distribution per sample ("per sample" column) and global feature distribution ("per feature").

	Per sample	Per feature
Min. frequency	1953	1
1st quartile	7920	9
Median frequency	14020	22
3rd quartile	22257	53
Max. frequency	75204	26456
Mean frequency	17061	193.25

The number of high quality sequence count ranges from 1953 to 75204, while feature abundance ranges from 1 to 26456 counts.

1.3. Sample-specific composition

Bar plot showing relative frequencies of the Top30 most abundant taxa across investigated samples: frequencies are shown at Family (QIIME2 "level 5") level. Feature quantification, taxonomic assignment and phylogenetic tree reconstruction were carried out through QIIME2 Suite (see Materials and Methods for details). Samples are ordered by class and sample names. Plot obtained through the "Taxonomic Bar Plots" application in QIIME2 website (https://view.qiime2.org/).

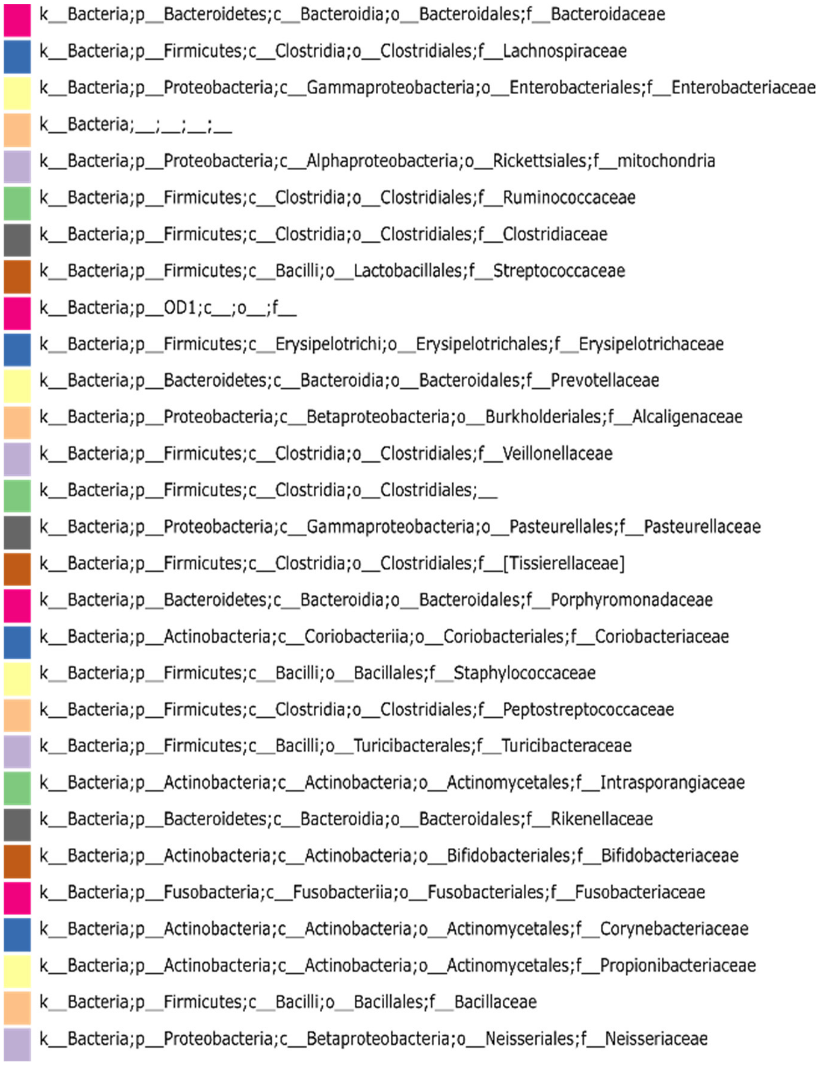

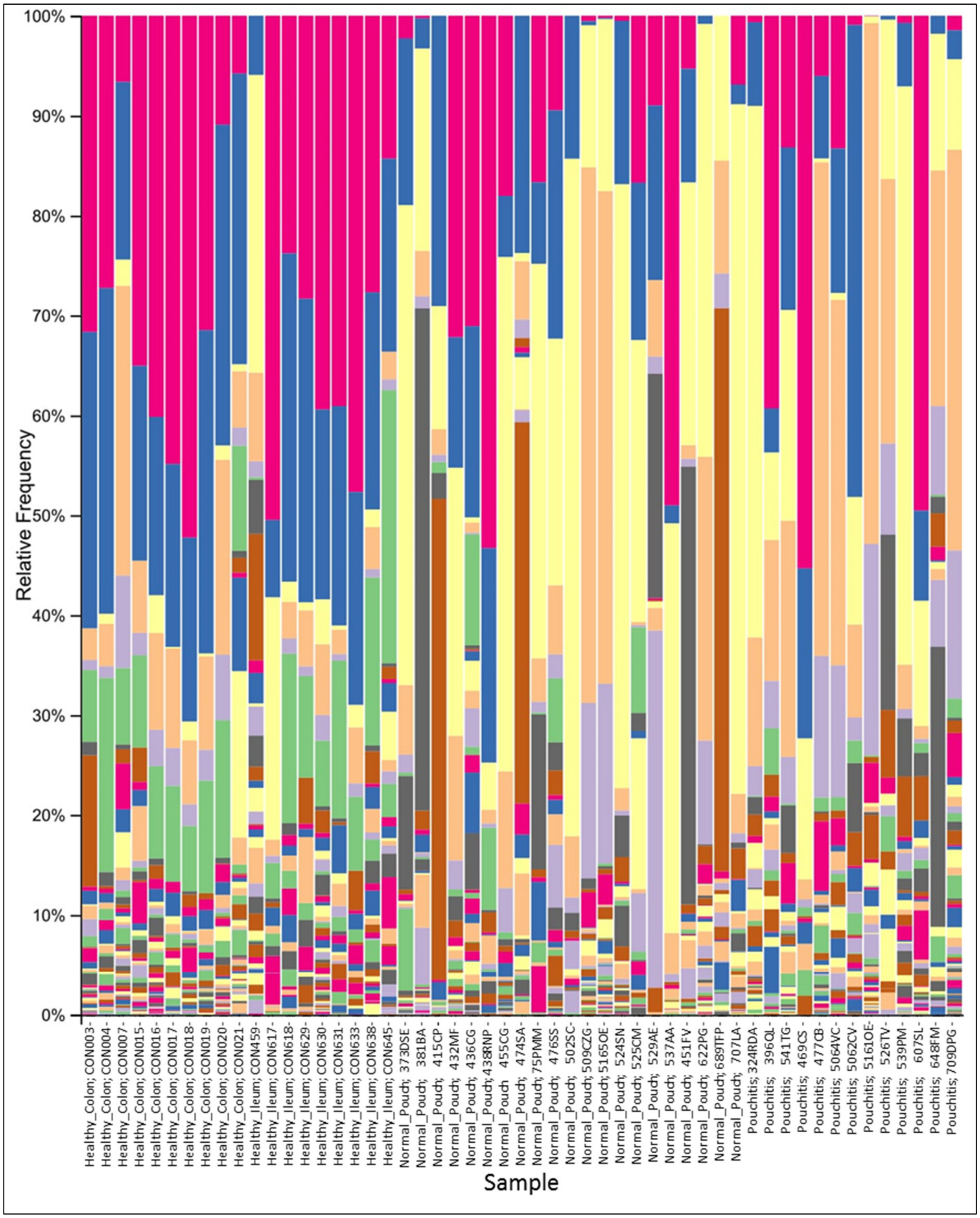

100%
90%
80%
70%
60%
50%
40%
30%
20%
10%
0%
Relative Frequency
Healthy_Colon; CON003
Healthy_Colon; CON004
Healthy_Colon; CON007
Healthy_Colon; CON015
Healthy_Colon; CON016
Healthy_Colon; CON017
Healthy_Colon; CON018
Healthy_Colon; CON019
Healthy_Colon; CON020
Healthy_Colon; CON021
Healthy_Ileum; CON459
Healthy_Ileum; CON617
Healthy_Ileum; CON618
Healthy_Ileum; CON629
Healthy_Ileum; CON630
Healthy_Ileum; CON631
Healthy_Ileum; CON633
Healthy_Ileum; CON638
Healthy_Ileum; CON645
Normal_Pouch; 373DSE
Normal_Pouch; 381BA
Normal_Pouch; 415CP
Normal_Pouch; 432MF
Normal_Pouch; 436CG
Normal_Pouch; 438RNP
Normal_Pouch 455CG
Normal_Pouch; 474SA
Normal_Pouch; 75PMM
Normal_Pouch; 476SS
Normal_Pouch; 502SC
Normal_Pouch; 509CZG
Normal_Pouch; 5165OE
Normal_Pouch; 524SN
Normal_Pouch; 525CM
Normal_Pouch; 529AE
Normal_Pouch; 537AA
Normal_Pouch; 451FV
Normal_Pouch; 622PG
Normal_Pouch; 689TFP
Normal_Pouch; 707LA
Pouchitis; 324RDA
Pouchitis; 396QL
Pouchitis; 541TG
Pouchitis; 469CS
Pouchitis; 477CB
Pouchitis; 5064VC
Pouchitis; 5062CV
Pouchitis; 5161OE
Pouchitis; 526TV
Pouchitis; 539PM
Pouchitis; 607SL
Pouchitis; 648FM
Pouchitis; 709DPG
Sample

1.4. Alpha diversity analysis.

We investigated Alpha diversity by calculating four different measures: Pielou's evenness (1.4.1), Faith's Phylogenetic Distance (1.4.2), Number of Observed Features (1.4.3) and Shannon's entropy (1.4.4). For each index, the following outcomes are provided: boxplot for group-specific index distribution (x-axis: 4 groups, "Healthy Colon", "Healthy Ileum", "Normal Pouch", "Pouchitis", y-axis: alpha-diversity index); Kruskal-Wallis test results (H statistics; p-value) for the global comparison of group-specific distribution; Kruskal-Wallis test results for pairwise group comparisons. Boxplot 1.4.4 is also reported in the main text, Figure 2. Analyses were performed through the "QIIME2 Diversity" module, using "4910" as cut-off for reads sampling depth (see main text for further details).

1.4.1-. Pielou's evenness

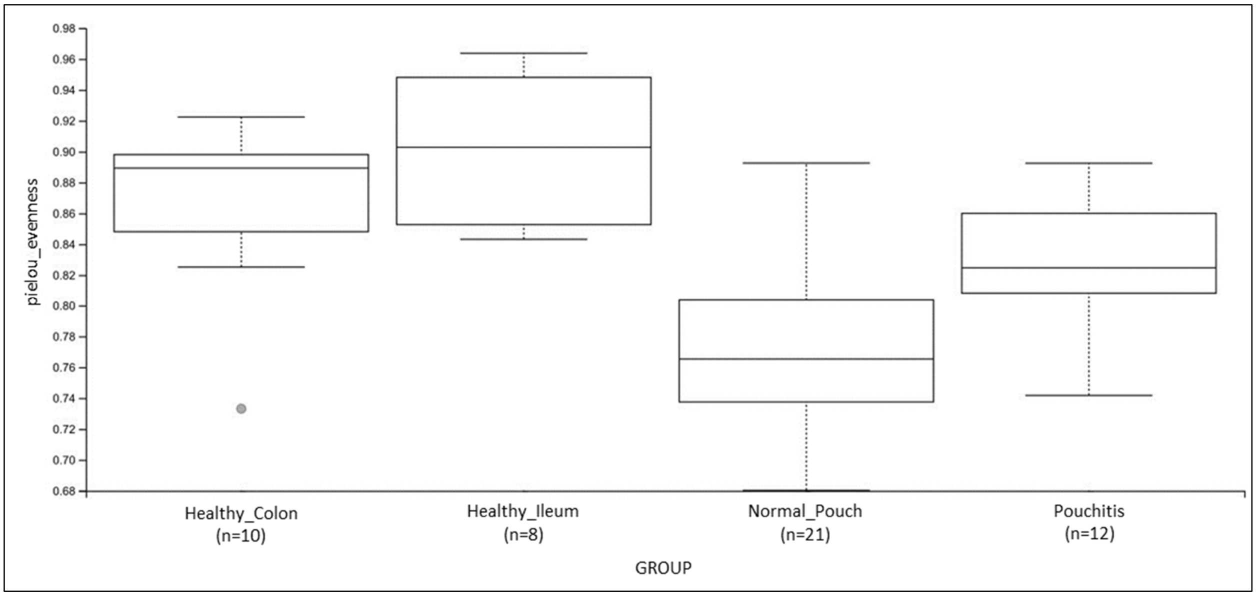

Kruskal-Wallis test (all groups): H = 23.94351; p-value= 0.00002

Kruskal-Wallis test (pairwise):

Group 1	Group 2	H	p-value	q-value
Healthy_Colon	Healthy_Ileum	1.13684	0.28632	0.28632
Healthy_Colon	Normal_Pouch	10.86428	0.00098	0.00294
Healthy_Colon	Pouchitis	4.73478	0.02955	0.03547
Healthy_Ileum	Normal_Pouch	13.75238	0.00021	0.00125
Healthy_Ileum	Pouchitis	7.71428	0.00547	0.01095
Normal_Pouch	Pouchitis	6.86274	0.0088	0.0132

1.4.2-. Faith's Phylogenetic Distance

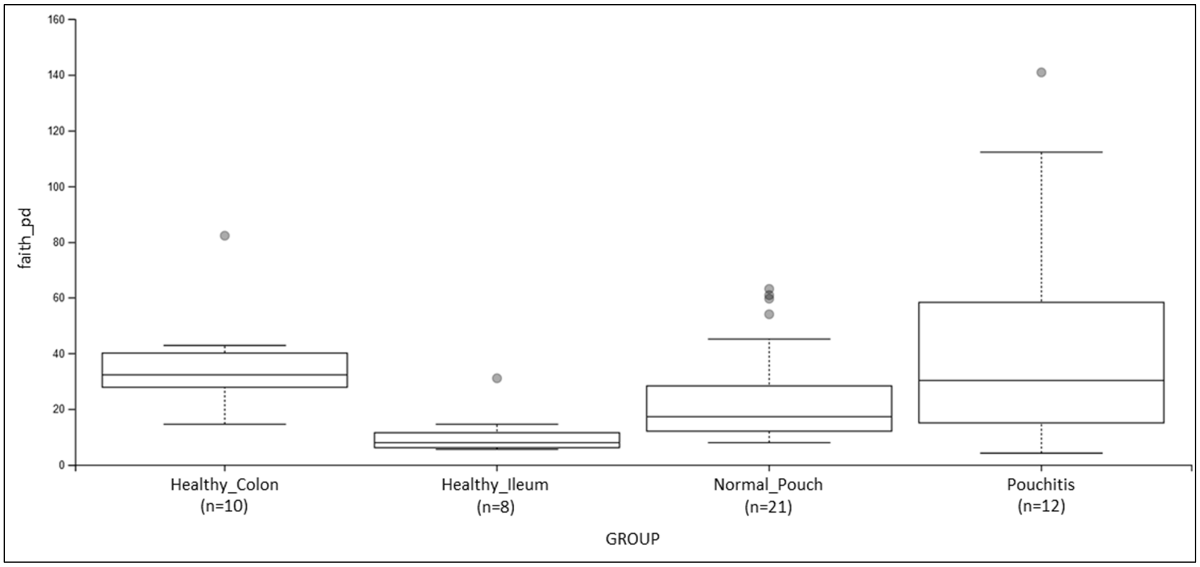

Kruskal-Wallis test (all groups): H = 13.28014; p-value= 0.00406
Kruskal-Wallis test (pairwise):

Group 1	Group 2	H	p-value	q-value
Healthy_Colon	Healthy_Ileum	10.23157	0.00138	0.00828
Healthy_Colon	Normal_Pouch	4.11428	0.04252	0.06378
Healthy_Colon	Pouchitis	0.03913	0.84319	0.84319
Healthy_Ileum	Normal_pouch	6.68809	0.00971	0.02179
Healthy_Ileum	Pouchitis	6.48214	0.01089	0.02179
Normal_Pouch	Pouchitis	1.17787	0.27778	0.33334

1.4.3-. Number of Observed Features

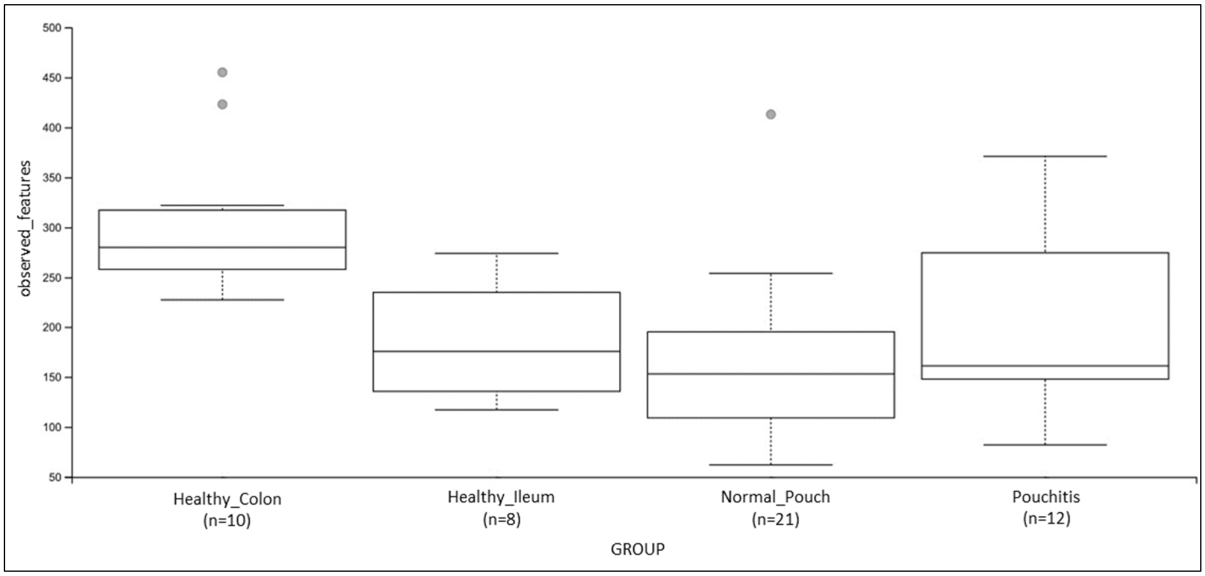

Kruskal-Wallis test (all groups): H = 16.67914; p-value= 0.00082
Kruskal-Wallis test (pairwise):

Group 1	Group 2	H	p-value	q-value
Healthy_Colon	Healthy_Ileum	8.08421	0.00446	0.01339
Healthy_Colon	Normal_Pouch	15.44775	8.48175e-05	0.00051
Healthy_Colon	Pouchitis	5.02608	0.02496	0.04993
Healthy_Ileum	Normal_Pouch	1.37176	0.24151	0.28981
Healthy_Ileum	Pouchitis	0.29166	0.58915	0.58915
Normal_Pouch	Pouchitis	1.61905	0.20322	0.28988

1.4.4-. Shannon entropy

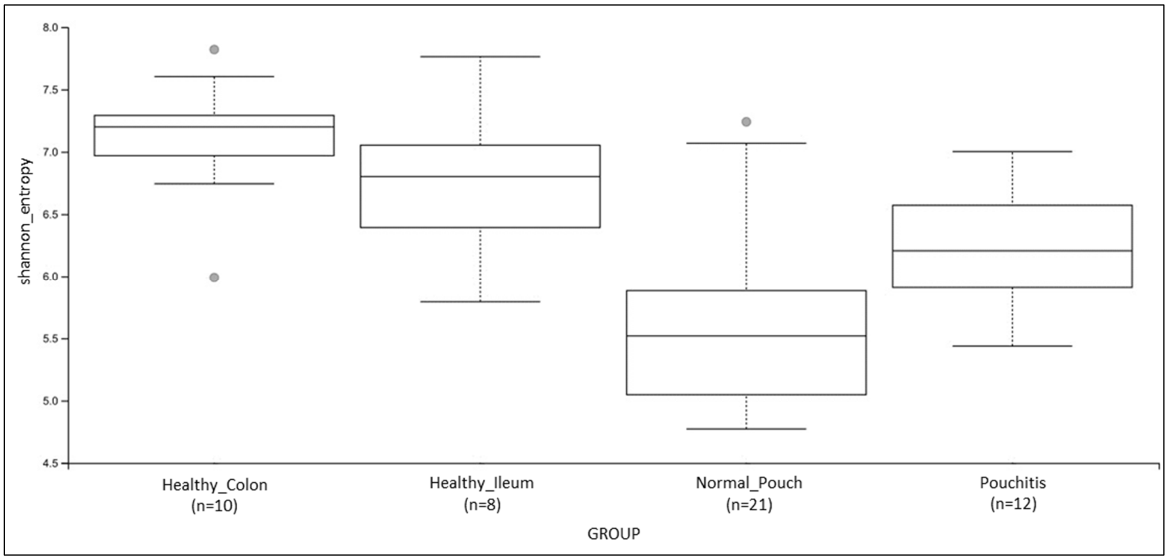

Kruskal-Wallis test (all groups): H = 25.10759; p-value= 0.00001
Kruskal-Wallis test (pairwise):

Group 1	Group 2	H	p-value	q-value
Healthy_Colon	Healthy_Ileum	2.02105	0.15513	0.15513
Healthy_Colon	Normal_Pouch	15.77857	7.1e-05	0.00042
Healthy_Colon	Pouchitis	10.43913	0.00123	0.00371
Healthy_Ileum	Normal_Pouch	9.15238	0.00248	0.00496
Healthy_Ileum	Pouchitis	2.38095	0.12282	0.14738
Normal_Pouch	Pouchitis	7.46358	0.00629	0.00944

1.5-. Beta diversity analysis.

Diversity among the four groups has been evaluated by considering four popular dissimilarity/distance methods: Bray-Curtis (1.5.1), Jaccard (1.5.2), Unweighted and Weighted Unifrac (1.5.3 and 1.5.4). For each method, the following outcomes are provided: a screenshot of three-dimensional Principal Coordinate Analysis ("3D-PCoA") plot; a table summarizing PERMANOVA test results for pairwise group comparisons.

PCoA plots have been obtained by using the Emperor web-application within QIIME2 website (https://view.qiime2.org/); red dots indicate "Healthy Colon" samples; blue: "Healthy Ileum"; orange for Normal "Pouch"; green for "Pouchitis". Furthermore, each table show details on comparing groups and their size, number of permutations, pseudo-F statistics, p-values and Benjamini-Hochberg adjusted p-value. PERMANOVA verifies the hypothesis that distances among samples with one class group differ from distances of these samples from samples of other groups.

PCoA plot obtained by using Jaccard distance is also reported in Main Text, as Figure 3.

1.5.1-. Bray-Curtis

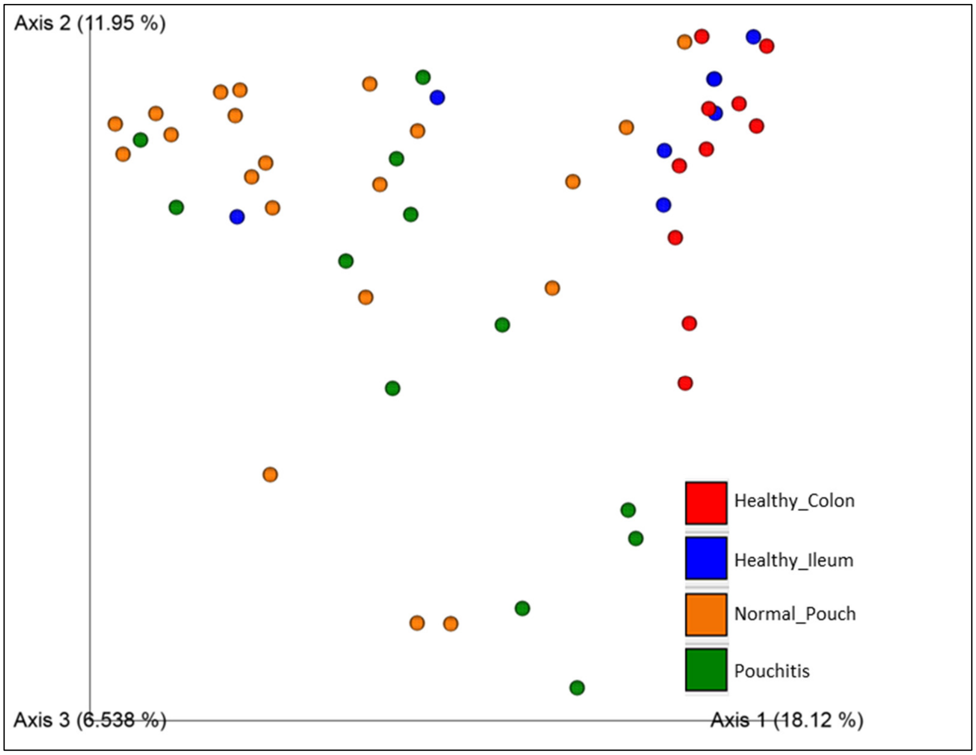

Group 1	Group 2	Sample size	Permutations	pseudo-F	p-value	q-value
Healthy_Colon	Healthy_Ileum	18	999	1.32284	0.096	0.096
Healthy_Colon	Normal_Pouch	31	999	5.31268	0.001	0.002
Healthy_Colon	Pouchitis	22	999	3.67678	0.001	0.002
Healthy_Ileum	Normal_Pouch	29	999	2.98257	0.003	0.00451
Healthy_Ileum	Pouchitis	20	999	2.43851	0.001	0.002
Normal_Pouch	Pouchitis	33	999	1.57663	0.076	0.09

1.5.2-. Jaccard

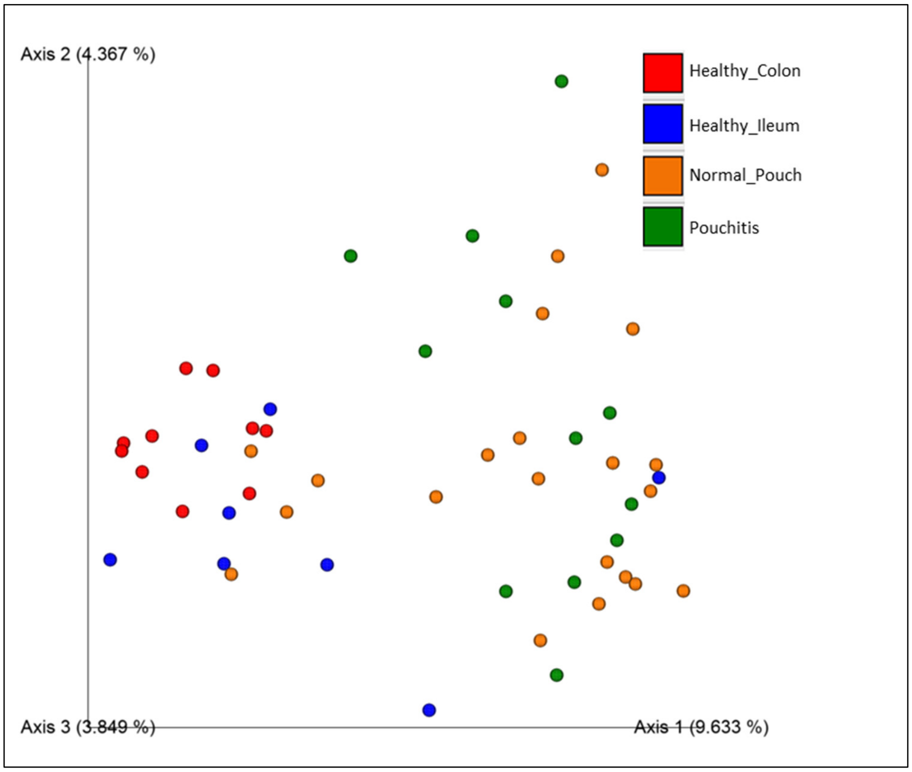

Group 1	Group 2	Sample size	Permutations	pseudo-F	p-value	q-value
Healthy_Colon	Healthy_Ileum	18	999	1.31431	0.019	0.02279
Healthy_Colon	Normal_Pouch	31	999	2.86588	0.001	0.0015
Healthy_Colon	Pouchitis	22	999	2.65488	0.001	0.0015
Healthy_Ileum	Normal_Pouch	29	999	1.73343	0.001	0.0015
Healthy_Ileum	Pouchitis	20	999	1.68959	0.001	0.0015
Normal_Pouch	Pouchitis	33	999	1.06149	0.239	0.239

1.5.3-. Unweighted Unifrac

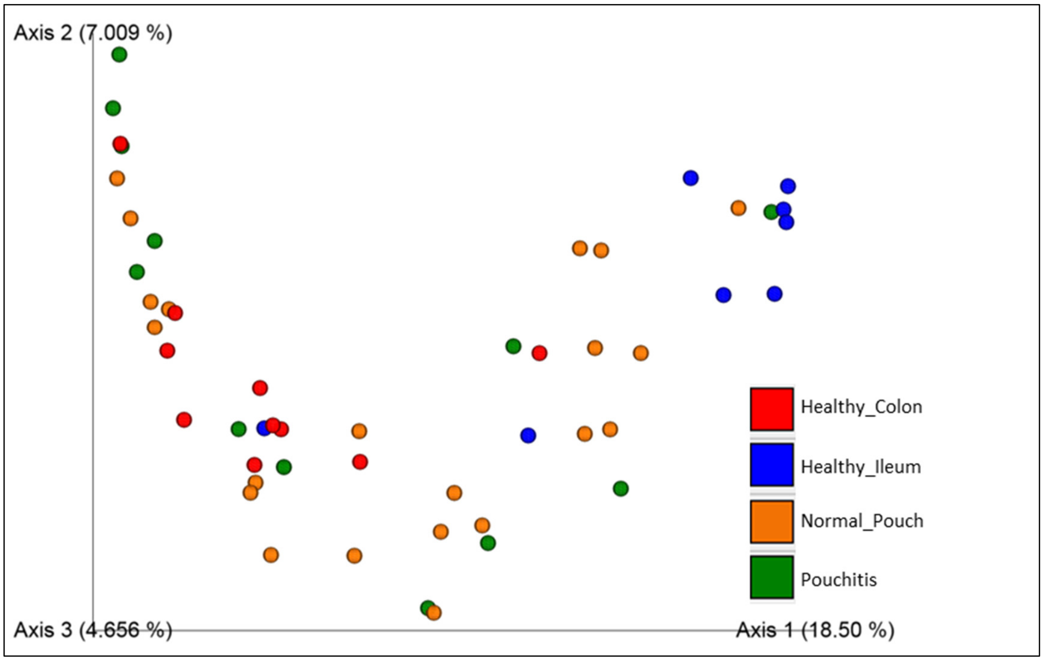

Group 1	Group 2	Sample size	Permuta-tions	pseudo-F	p-value	q-value
Healthy_Colon	Healthy_Ileum	18	999	4.43671	0.003	0.006
Healthy_Colon	Nor-mal_Pouch	31	999	1.80997	0.003	0.006
Healthy_Colon	Pouchitis	22	999	1.47816	0.053	0.0636
Healthy_Ileum	Nor-mal_Pouch	29	999	2.71733	0.002	0.006
Healthy_Ileum	Pouchitis	20	999	2.90128	0.008	0.012
Nor-mal_Pouch	Pouchitis	33	999	0.95126	0.496	0.496

1.5.4-. Weighted Unifrac

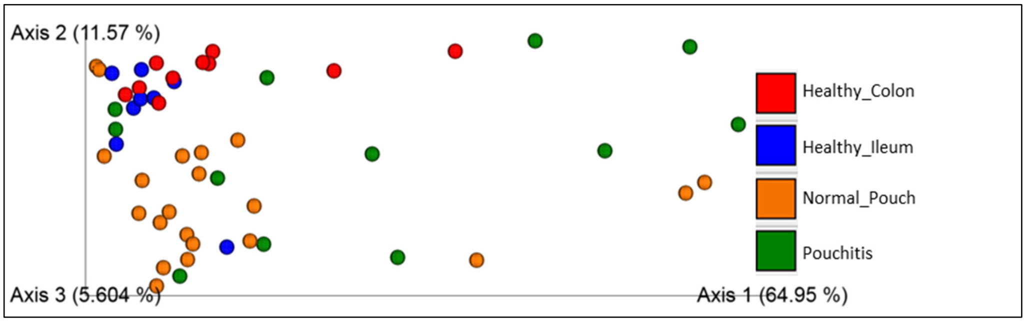

Group 1	Group 2	Sample size	Permutations	pseudo-F	p-value	q-value
Healthy_Colon	Healthy_Ileum	18	999	2.73618	0.025	0.0375
Healthy_Colon	Normal_Pouch	31	999	3.91585	0.013	0.0375
Healthy_Colon	Pouchitis	22	999	4.02731	0.022	0.0375
Healthy_Ileum	Normal_Pouch	29	999	2.61983	0.052	0.052
Healthy_Ileum	Pouchitis	20	999	5.63246	0.006	0.036
Normal_Pouch	Pouchitis	33	999	3.53156	0.04	0.048

1.6. ANCOM

QIIME2 ANCOM module was used to infer microbial genera or species that are differentially abundant across sample groups. Rare features and mitochondrial/plastid sequences were removed from the feature table (see Methods). Thus, features were collapsed according to QIIME2 taxonomical classification (based on GreenGenes resource) into "genus-collapsed" ("level 6") and "species-collapsed" ("level 7") features, for a total of, respectively, 66 and 95 features. For both strategies, ANCOM Volcano Plot and statistical results table are provided.

Significant features are placed on the top-right corner of the Volcano Plot; the first table shows significant features together with the corresponding W statistics, i.e., the number of sub-hypotheses that have passed for a certain feature (ANCOM compares pairs of feature relative abundances). The second table shows the percentile abundance of relevant features across the sample groups. For example, a value of 100 at 50% percentile indicates that the detected feature has a maximum sequence count of 100 in the 50% of samples of an investigated group.

1.6.1-. Results for genus-collapsed features (N = 66)

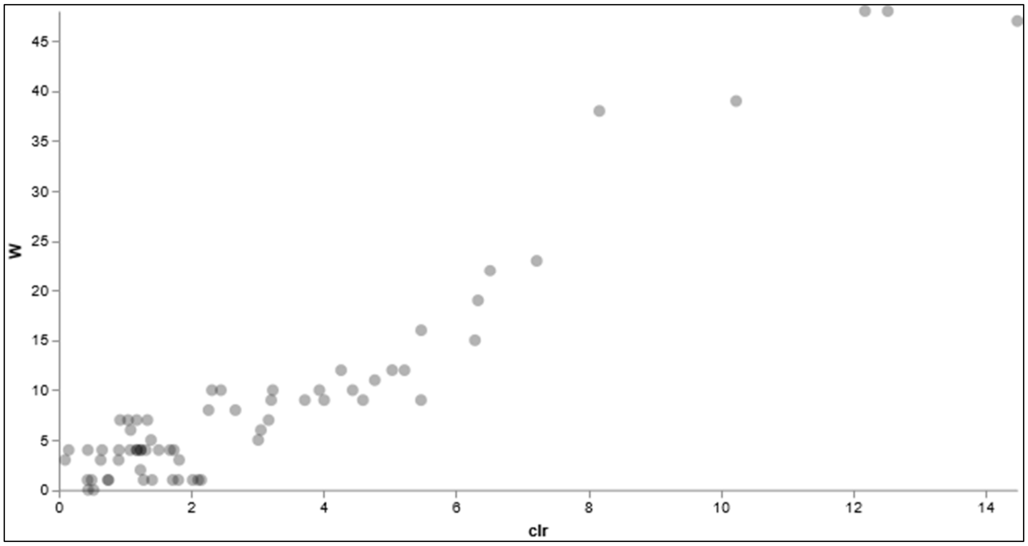

Feature	W
k__Bacteria;p__Firmicutes;c__Clostridia;o__Clostridiales;f__Ruminococcaceae;g__Faecalibacterium	48
k__Bacteria;p__Proteobacteria;c__Gammaproteobacteria;o__Enterobacteriales;f__Enterobacteriaceae;__	48
k__Bacteria;p__Firmicutes;c__Clostridia;o__Clostridiales;f__Ruminococcaceae;g__Gemmiger	47

Percentile	Group	k__Bacteria;p__Firmicutes;c__Clostridia;o__Clostrid-ial-es;f__Ruminococcaceae;g__Faecalibacterium	k__Bacteria;p__Firmicutes;c__Clostridia;o__Clostridiales;f__Ruminococcaceae;g__Gemmiger	k__Bacteria;p__Proteobacteria;c__Gammaproteobacteria;o__Enterobacteriales;f__Enterobacteriaceae;__
	**W**	**48**	**47**	**48**
0.0	Healthy_Colon	643.0	68.0	1.0
25.0	Healthy_Colon	784.75	130.5	25.75
50.0	Healthy_Colon	1007.0	259.0	120.5
75.0	Healthy_Colon	1348.75	372.75	546.25
100.0	Healthy_Colon	2535.0	1438.0	688.0
0.0	Healthy_Ileum	1.0	1.0	1.0
25.0	Healthy_Ileum	227.0	92.0	40.0
50.0	Healthy_Ileum	231.0	144.0	123.0
75.0	Healthy_Ileum	621.0	222.0	743.0
100.0	Healthy_Ileum	1092.0	279.0	2090.0
0.0	Normal_Pouch	1.0	1.0	1.0
25.0	Normal_Pouch	1.0	1.0	2686.0
50.0	Normal_Pouch	1.0	1.0	4520.0
75.0	Normal_Pouch	59.0	47.0	5465.0
100.0	Normal_Pouch	1337.0	843.0	14689.0
0.0	Pouchitis	1.0	1.0	62.0
25.0	Pouchitis	1.0	1.0	312.0
50.0	Pouchitis	1.0	1.0	1172.0
75.0	Pouchitis	91.0	1.0	2577.0
100.0	Pouchitis	269.0	311.0	7331.0

1.6.2-. Results for species-collapsed features (N=95)

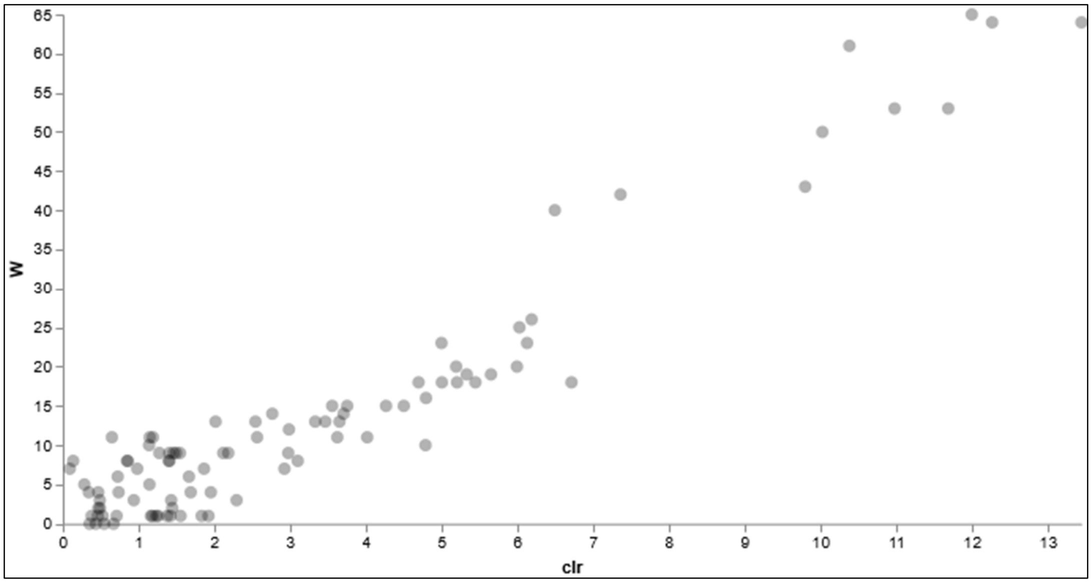

Feature	W
k__Bacteria;p__Firmicutes;c__Clostridia;o__Clostridiales;f__Ruminococcaceae;g__Faecalibacterium;s__prausnitzii [A]	65
k__Bacteria;p__Proteobacteria;c__Gammaproteobacteria;o__Enterobacteriales;f__Enterobacteriaceae;__;__ [B]	64
k__Bacteria;p__Firmicutes;c__Clostridia;o__Clostridiales;f__Ruminococcaceae;g__Gemmiger;s__formicilis [C]	64
k__Bacteria;p__Firmicutes;c__Clostridia;o__Clostridiales;f__Lachnospiraceae;g__Roseburia;__ [D]	61
k__Bacteria;p__Firmicutes;c__Clostridia;o__Clostridiales;f__Lachnospiraceae;g__Blautia;s__obeum [E]	53
k__Bacteria;p__Firmicutes;c__Clostridia;o__Clostridiales;f__Lachnospiraceae;g__[Ruminococcus];s__torques [F]	53
k__Bacteria;p__Bacteroidetes;c__Bacteroidia;o__Bacteroidales;f__Porphyromonadaceae;g__Parabacteroides;s__ [G]	50
k__Bacteria;p__Firmicutes;c__Clostridia;o__Clostridiales;f__Lachnospiraceae;g__Dorea;s__formicigenerans [H]	43
k__Bacteria;p__Bacteroidetes;c__Bacteroidia;o__Bacteroidales;f__Bacteroidaceae;g__Bacteroides;s__uniformis [I]	42

Percentile	Group	[A]	[B]	[C]	[D]	[E]	[F]	[G]	[H]	[I]
	**W**	**65**	**64**	**64**	**61**	**53**	**53**	**50**	**43**	**42**
0.0	Healthy_Colon	643.0	68.0	1.0	314.0	1.0	1.0	1.0	1.0	1.0
25.0	Healthy_Colon	784.75	130.5	25.75	349.75	53.0	77.0	68.5	67.5	65.25
50.0	Healthy_Colon	1007.0	259.0	120.5	496.5	86.5	98.0	82.5	113.5	143.0
75.0	Healthy_Colon	1348.75	372.75	546.25	850.75	111.25	141.0	535.5	178.0	181.25
100.0	Healthy_Colon	2535.0	1438.0	688.0	1642.0	1809.0	208.0	931.0	1086.0	394.0
0.0	Healthy_Ileum	1.0	1.0	1.0	1.0	1.0	1.0	1.0	1.0	1.0
25.0	Healthy_Ileum	227.0	92.0	40.0	67.0	1.0	1.0	51.0	1.0	32.0
50.0	Healthy_Ileum	231.0	144.0	123.0	138.0	42.0	55.0	83.0	60.0	39.0
75.0	Healthy_Ileum	621.0	222.0	743.0	253.0	60.0	88.0	97.0	68.0	54.0
100.0	Healthy_Ileum	1092.0	279.0	2090.0	350.0	73.0	130.0	154.0	174.0	86.0

0.0	Normal_Pouch	1.0	1.0	1.0	1.0	1.0	1.0	1.0	1.0	1.0
25.0	Normal_Pouch	1.0	1.0	2686.0	1.0	1.0	1.0	1.0	1.0	1.0
50.0	Normal_Pouch	1.0	1.0	4520.0	1.0	1.0	1.0	1.0	1.0	1.0
75.0	Normal_Pouch	59.0	47.0	5465.0	92.0	1.0	1.0	1.0	26.0	1.0
100.0	Normal_Pouch	1337.0	843.0	14689.0	1124.0	144.0	118.0	208.0	112.0	277.0
0.0	Pouchitis	1.0	1.0	62.0	1.0	1.0	1.0	1.0	1.0	1.0
25.0	Pouchitis	1.0	1.0	312.0	1.0	1.0	1.0	1.0	1.0	1.0
50.0	Pouchitis	1.0	1.0	1172.0	1.0	1.0	1.0	1.0	1.0	1.0
75.0	Pouchitis	91.0	1.0	2577.0	57.0	1.0	1.0	1.0	1.0	1.0
100.0	Pouchitis	269.0	311.0	7331.0	533.0	1.0	1.0	31.0	1.0	235.0
